# Evaluation of the taste-masking effects of (2-hydroxypropyl)-β-cyclodextrin on ranitidine hydrochloride; a combined biosensor, spectroscopic and molecular modelling assessment†

**DOI:** 10.1039/c7ra11015d

**Published:** 2018-01-17

**Authors:** Sai Kin Chay, Alison V. Keating, Colin James, Abil E. Aliev, Shozeb Haider, Duncan Q. M. Craig

**Affiliations:** University College London School of Pharmacy 29-39 Brunswick Square London WC1N 1AX UK duncan.craig@ucl.ac.uk; University College London Department of Chemistry 20 Gordon Street London WC1H 0AJ UK

## Abstract

Taste assessment in an increasingly important aspect of formulation development, particularly for paediatric medications. Electronic taste sensing systems have the potential to offer a rapid, objective and safe method of taste assessment prior to the use of more costly human panels or animal models. In this study, the ability of the TS-5000Z taste sensing system to assess the taste masking efficiency of (2-hydroxypropyl)-β-cyclodextrin (HP-β-CyD) complexes with ranitidine hydrochloride was evaluated in order to explore the potential of the biosensor approach as a means of assessing taste masking by inclusion complexation. Nuclear magnetic resonance (NMR) spectroscopy and molecular docking studies were employed to identify and examine the interaction between ranitidine hydrochloride and HP-β-CyD. Taste-masking efficiencies were determined by the Euclidean distance between taste-masked formulations and the pure drug substance on a PCA score plot. The results showed that with increasing molarity of HP-β-CyD in the formulation, the distance from ranitidine hydrochloride increased, thus indicating a significant difference between the taste of the formulation and that of the pure drug. NMR studies also provided strong supporting evidence for the complexation between HP-β-CyD and ranitidine hydrochloride, with the H3′ region of the former identified as the most likely binding site for the drug. Molecular docking studies suggested that the dimethylamino and diamine groups of the drug form direct hydrogen bonds with the hydroxyl oxygen atoms of HP-β-CyD, while the furan ring docks in close proximity to H3′. This study has demonstrated that the biosensor system may provide quantitative data to assess bitterness of inclusion complexes with HP-β-CyD, while spectroscopic and modelling studies may provide a mechanistic explanation for the taste masking process. This in turn suggests that there is a role for biosensor approaches in providing early screening for taste masking using inclusion complexation and that the combination with mechanistic studies may provide insights into the molecular basis of taste and taste masking.

## Abstract 2

A correlation between taste masking, assessed using a taste sensor system, and inclusion complex formation, assessed using NMR and molecular docking studies, has been identified for ranitidine HCl and (2-hydroxypropyl)-β-cyclodextrin.

## Graphical abstract

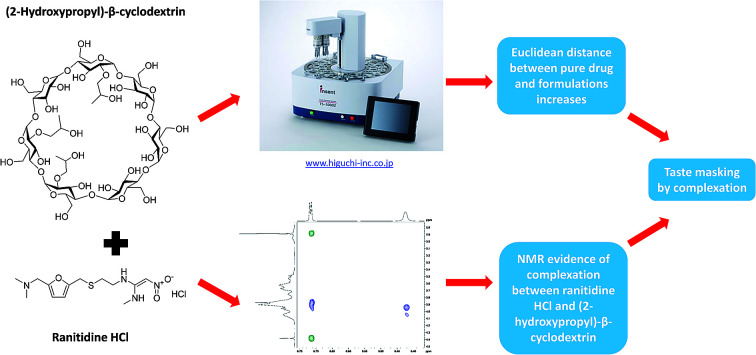

## Introduction

Taste is often a key factor in the development of pharmaceutical formulations, particularly for paediatric and elderly patients, due to the associated direct influence on patient adherence.^1^ The five specific tastes include saltiness, sourness, bitterness, sweetness and umami; bitterness is the most problematic for pharmaceutical formulations due to its intuitive association with toxicity. The sensory mechanism of signal transduction following binding of a molecule to a taste receptor varies depending on the taste in question. For example, saltiness is mediated by sodium ion flux through apical sodium channels, sourness is mediated through a hydrogen ion blockade of potassium or sodium channels while sweetness and bitterness are transmitted *via* G protein-coupled receptors,^2^ with the perception of bitterness being mediated by bitter taste receptors (T2Rs) in the oral cavity.^3^

**Fig. 1:**
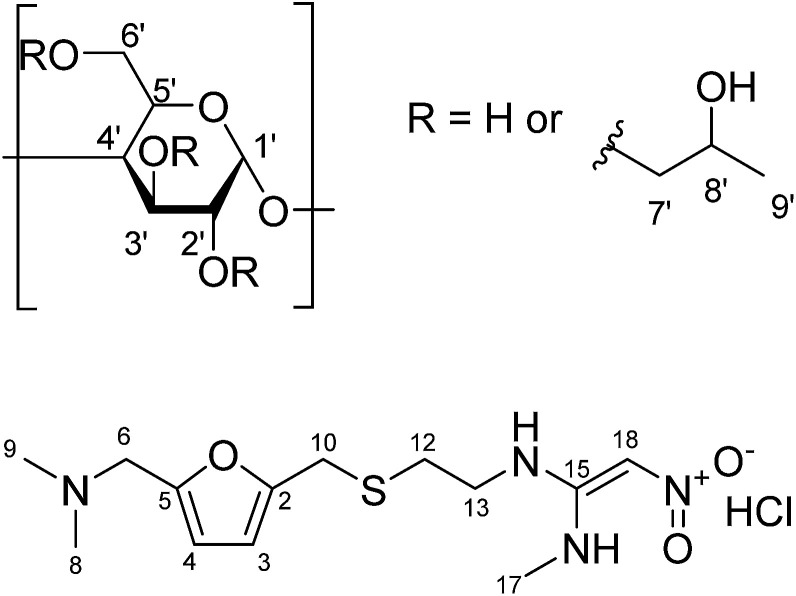
Chemical structure of (a) (2-hydroxypropyl)-β-cyclodextrin (with protons numbered); (b) ranitidine HCl.

Ranitidine hydrochloride (Fig. 1) is an H_2_-receptor antagonist that reduces acid production in the stomach and is commonly used to treat gastrointestinal diseases such as duodenal ulcer, reflux oesophagitis and Zollinger–Ellison Syndrome. It is included on the World Health Organisation's List of Essential Medicines which specifies medicines required for basic healthcare.^4^ However, ranitidine hydrochloride is also known to have a bitter taste and a sulphur-like odour.^5^ These may potentially be significant barriers to patient adherence, hence the exploration of taste-masking approaches is of relevance for this drug.

There are various taste-masking techniques which may be used to inhibit bitter taste.^6^ For solid oral dosage forms, polymer coating of capsules and tablets or monolithic systems such as polymer or lipid extrudates may be used;^7^ these approaches may be of less use for paediatric patients for whom swallowing solid dosage forms can be challenging. Liquid formulation approaches may include chemical modifications to the drug, microencapsulation, or simply the addition of taste masking excipients such as sugars, sweeteners and sweetness enhancers.^8^ Complexation by cyclodextrins or ion exchange resins can also be considered. Cyclodextrins (CDs) belong to a class of cyclic oligosaccharides composed of α-d-glycopyranose units linked by α-1,4 glycosidic bonds. The α-, β- and γ-cyclodextrins are widely used examples of, with six, seven and eight d-glycopyranose units, respectively. Cyclodextrins exhibit a central cavity lined with carbon and oxygen atoms of the glucose residues and a hydrophilic exterior made up of alcoholic hydroxyl groups. As a result, cyclodextrins possess the ability to include guest molecule inside their cavities, forming inclusion complexes. These inclusion complexes can enhance drug solubility, mask bitter taste of the active pharmaceutical ingredient (API) and prevent degradation of drug molecules.^9^ (2-Hydroxypropyl)-β-cyclodextrin (HP-β-CyD), a hydroxyalkyl derivative of β-CD (Fig. 1), was introduced to improve aqueous solubility of β-CD as well as to enhance inclusion capability.^10^ This chemically modified derivative of β-CD has also displayed an enhanced safety profile compared to its parent compound.^11^

The three main approaches to taste-masking assessment are human taste panels, animal models and *in vitro* analytical techniques. Taste assessment by a human panel is challenging with regards to cost, time and unknown toxicity status of new drug entities. Moreover, the approach is susceptible to variations in the physiology of both the various patient populations and the individuals concerned. Animal models such as the rodent BATA model have also shown great promise in assessing the taste of APIs with comparable results to human taste panel data, however further validation work is still needed.^12,13^*In vitro* analytical techniques include the use of dissolution testing and electronic taste sensing systems. Dissolution testing detects the amount of free drug in a solution as a function of time but is a crude indication of the experience of taste.^14^ Electronic taste sensing systems are composed of multichannel taste sensors, involving a series of lipid/polymer membrane coatings.^15^ These multichannel sensors measure a pattern of signals associated with the membrane potentials which may then be associated with taste quality.^16,17^ These systems therefore have the potential to offer an objective and safe method for taste-masking assessment, although validation remains a matter of ongoing investigation.^18^ The two most commonly used, commercially available taste-sensing systems are Alpha Astree II (Alpha MOS, Toulouse, France) and TS-5000Z (Insent Inc., Atsugi-shi, Japan); in this study, the latter was used. The taste sensing system is made up of a working electrode with a lipid/polymer membrane used to detect taste substances, a handle and a data processing unit.

The system is designed to detect taste in a similar manner to human gustatory sensation where lipid/polymer membranes, transducer and statistical analysis in the system mirror the taste buds, neural transmission and cognition in the thalamus in humans respectively. Taste substances cause changes in electric charge density of the lipid/membrane surface and/or ion distribution near the surface of the membrane.^19^ The total electric change is then given as the response membrane electric potential for the substances. While most studies exploring the use of taste sensors for pharmaceuticals have focused on assessing the quantitative response for particular drug systems, there is an emerging emphasis on looking at how the approach may be used to assess taste masking strategies. In particular, a previous investigation has compared the ability of the e tongue approach with an animal model to assess cyclodextrin as a means of taste masking praziquantel.^20^

The rationale for using an electronic taste sensing system to determine taste-masking effects is that it enables one to measure the activity of molecules within a formulation based on its non-specific multi-sensor approach. In addition, the sensors are able to interact with different chemical structures, which allows one to assume an overall impression rather than to determine the concentration of the drug substance. If a (log-linear) dependency between concentrations of the bitter tasting drug substance and the sensor response exists, good results for the determination of taste-masking properties can be expected. Since the underlying measurement principle is potentiometric, sensor responses will most likely be derived from charged molecules. Therefore, it is reasonable to deduce that any reduction in sensor response is based on a lower exposure to the drug substance due to the taste-masking approach in question.^21^

In this study we have explored the potential of the approach to recognize the role of inclusion complexation as a means of masking taste in relation to the complexation process itself. In particular, we evaluate the effectiveness of HP-β-CyD to reduce the (measured) bitterness of ranitidine hydrochloride in relation to the complex formation process, as assessed using nuclear magnetic resonance spectroscopy (NMR); we also investigate molecular docking studies of ranitidine complexation with HP-β-CyD in order to obtain some basic information on the likely configuration of any such complex. In this manner we intend to correlate (or otherwise) the measured assessment of taste with the structure of the associated complex, thereby facilitating the use of the biosensor approach to provide an early, inexpensive evaluation of complex-based taste-masking approaches.

## Materials and methods

### Materials

Ranitidine hydrochloride, (2-hydroxypropyl)-β-cyclodextrin, quinine hydrochloride dihydrate, potassium chloride, tartaric acid, potassium hydroxide, hydrochloric acid (32%) were purchased from Sigma-Aldrich Chemie GmbH (Steinheim, Germany). Absolute ethanol was purchased from Fisher Chemical (Geel, Belgium). The inner solution (3.33 mol l^−1^ potassium chloride in saturated silver chloride) for sensors and reference electrodes of the taste sensing system was provided by Insent Inc. (Atsugi-shi, Japan).

### Electronic tongue measurements

The Insent TS-5000Z electronic tongue (Insent Inc., Atsugi-shi, Japan) was equipped with four lipid membrane sensors and two corresponding reference electrodes; three represent bitterness (SB2AC0, SB2AN0 and SB2C00) and one represents astringency (SB2AE1). SB2AC0 is dedicated to bitter cationic substances, SB2AN0 is dedicated to bitter cationic and neutral substances while SB2C00 is dedicated to bitter anionic substances. Each of these sensors was filled with 0.2 ml of inner solution, while the reference electrodes were filled with 0.4 ml of inner solution. All sensors were immersed in standard solution (see below) for 24 hours as preconditioning before measurement.

Two washing solutions for negatively and positively charged sensors were prepared respectively. For the negatively charged sensors, 30% ethanol in distilled water with 100 mM of hydrochloric acid was used, while for the positively charged sensors, 100 mM of potassium chloride with 10 mM potassium hydroxide were added to 30% ethanol. The standard solution serving as cleaning and reference solution was prepared by dissolving 0.3 mM tartaric acid and 30 mM potassium chloride in distilled water.

Sensor checks were carried out before every measurement to ensure the sensors were working in the correct mV range; each sample was measured four times. The first run was discarded as recommended by the supplier to allow for sensor conditioning. The assessment cycle consisted of measuring the reference solution (*V*_r_), the sample solution (*V*_s_), a short cleaning procedure (2 × 3 s), measurement of the after taste (*V*_r′_) and finally followed by a cleaning procedure for 330 s. The sensor output for taste, also called relative value (*R*) was determined with respect to the initial measured sensor response to the reference solution (*V*_r_).

1 *R* = *V*_s_ − *V*_r_

### Test solutions

Six different concentrations of ranitidine hydrochloride solutions, (between 0.06 mg ml^−1^ and 1.50 mg ml^−1^) were prepared in distilled water. Quinine hydrochloride dihydrate (molecular mass = 396.91 g mol^−1^) and HP-β-CyD (molecular mass = ∼1380 g mol^−1^) solutions were prepared in the same molarity as the ranitidine hydrochloride (molecular mass = 350.86 g mol^−1^) solutions so to allow for better comparability (Table S1†).

Five taste-masked formulations with different molar ratios of ranitidine hydrochloride to HP-β-CyD (1 : 1, 1 : 2, 1 : 3, 1 : 4 and 1 : 5) were prepared by mixing ranitidine hydrochloride powder with increasing amount of HP-β-CyD. The concentrations of HP-β-CyD in each of these taste-masked formulations are given in Table S2.†

### Data analysis

In the assessment of taste-masking properties, the sensor signals of the taste-masked formulations are compared with that of the pure drug solution using principal component analysis (PCA). PCA aims to summarize and reduce multidimensional data by transforming sensor signals to principal components, in order to describe the measured formulation samples in a new space with fewer dimensions. The PCA map is presented in a two-dimensional graph with the discriminating factors as principal component 1 (PC1) on the *x*-axes and principal component 2 (PC2) on the *y*-axes. As a result, the PCA map can be evaluated visually by the location of the measured samples. Since the pure drug solution exhibits a known unpleasant bitter taste, the larger the distance between the pure drug and the formulations, the greater the difference in taste. This distance between measured samples, known as Euclidean distances, can be calculated by equation shown in (2), where p and q represent the samples and *n* is the number of variables used for the model.^22^ Data processing, graphical representation and statistical interpretation of results were performed using OriginPro 2016 (OriginLab, Northampton, MA, USA).2
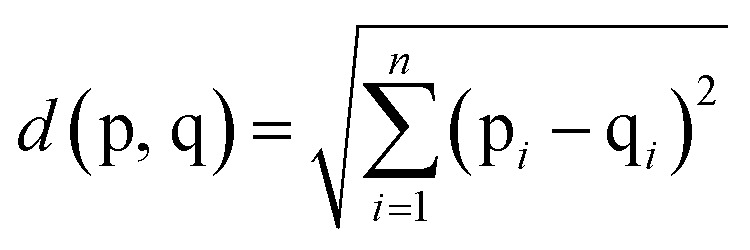


### NMR studies

Solutions of samples were prepared in 99.96% D_2_O (Cambridge Isotope Laboratories). In the first instance, ^1^H-NMR spectra of HP-β-CyD and ranitidine hydrochloride were recorded separately. In order to examine the complexation process between ranitidine hydrochloride and HP-β-CyD in solution, two stock solutions of 40 mM were prepared. Based on these two equimolar solutions, three samples containing both ranitidine hydrochloride and HP-β-CyD were prepared. This was achieved by mixing the two solutions at varying proportions, so that a range (0 < *r* < 1) of the ratio *r* = [X]/([H] + [G]) was sampled.^23^ In this experiment X = [H] and [H] and [G] are the total concentrations of the host (HP-β-CyD) and guest (ranitidine hydrochloride), respectively. Therefore, the total concentration [H] + [G] = [M] = 40 mM was kept constant for each solution. Table S3† illustrates the preparation process.

Solution ^1^H and ^13^C NMR spectra were recorded on a Bruker Avance III 600 MHz NMR spectrometer equipped with a 5 mm cryoprobe (^1^H 600.13 MHz and ^13^C 150.90 MHz). Data acquisition and processing were performed using standard TopSpin (version 3.2) software. ^1^H and ^13^C chemical shifts were calibrated using dioxane shifts in D_2_O (^1^H 3.75 ppm, ^13^C 67.19 ppm). NMR measurements were carried out at 298 K. A set of 2D experiments were used for assignment of ^1^H and ^13^C signals, including COSY for ^1^H–^1^H correlations *via* proton *J*_HH_ couplings, NOESY for ^1^H–^1^H correlations *via* NOEs (nuclear Overhauser effects) and HSQC for ^1^H–^13^C correlations *via* one-bond ^1^*J*_CH_ couplings. Standard Bruker pulse sequences *seldigpzs* and *selcssfdizs* were used for acquisition of selective 1D TOCSY spectra.

### Molecular modelling

The structure of HP-β-CyD was downloaded from the Protein Data Bank (code 2y4s) and treated as the receptor molecule. The structure of ranitidine was sketched in LigEdit module, charges assigned and docked using the ICM-Pro Molecular Modelling Suite (http://www.molsoft.com). Grid maps of size 15 × 15 × 10 Å^3^ were generated that encompassed the entire central cavity of β-cyclodextrin structure. Docking was run with an effort of 5, storing all alternative conformations of the ligand. A maximum of 25 docked conformations were generated. The final conformation was chosen based on strongest interaction energy between HP-β-CyD and ranitidine. Visualization of the docked poses was done using Pymol (http://www.pymol.org) and ICM-Pro Molsoft molecular modelling package.

## Results and discussion

### Assessment of ranitidine hydrochloride and HP-β-CyD alone

**Fig. 2:**
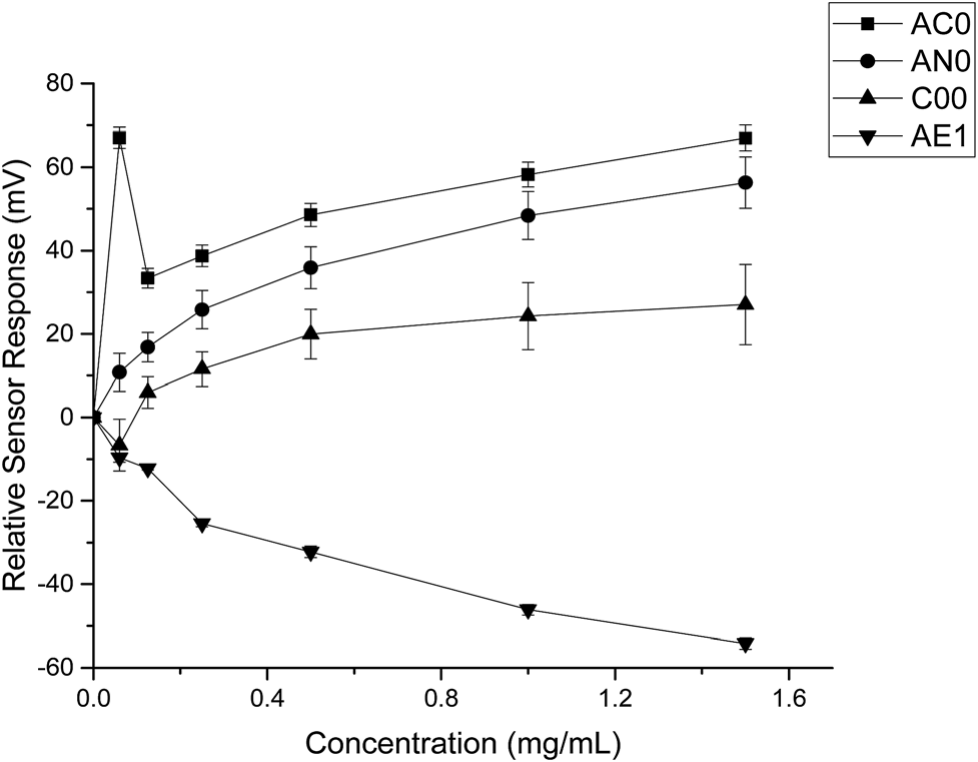
Sensor response curve for ranitidine hydrochloride showing normalised sensor response as a function of concentration (*n* = 3, mean ± S.D.).

The taste sensing system was first used to quantify the sensor response for ranitidine hydrochloride as a function of concentration (Fig. 2). All four sensors presented clear dependency between concentrations of the drug substance and sensor responses; it was also observed that the three bitterness sensors (SB2AC0, SB2AN0 and SB2C00) displayed a positive correlation whereas the astringency sensor SB2AE1 exhibited a negative correlation between the two variables. However, it is noticeable at 0.06 mg ml^−1^ (0.171 mM), sensor output from SB2AC0 spiked to its maximum before decreasing and following a concentration-dependent response. Similar observation could be observed for SB2C00 where sensor output dropped initially at 0.06 mg ml^−1^ (0.171 mM) before rising again and assuming a logarithmic relationship. Previous authors^24^ have suggested that the relationship between concentration and sensor response may be complex and dependent on both the sensor and the substrate on a case by case basis.

**Fig. 3:**
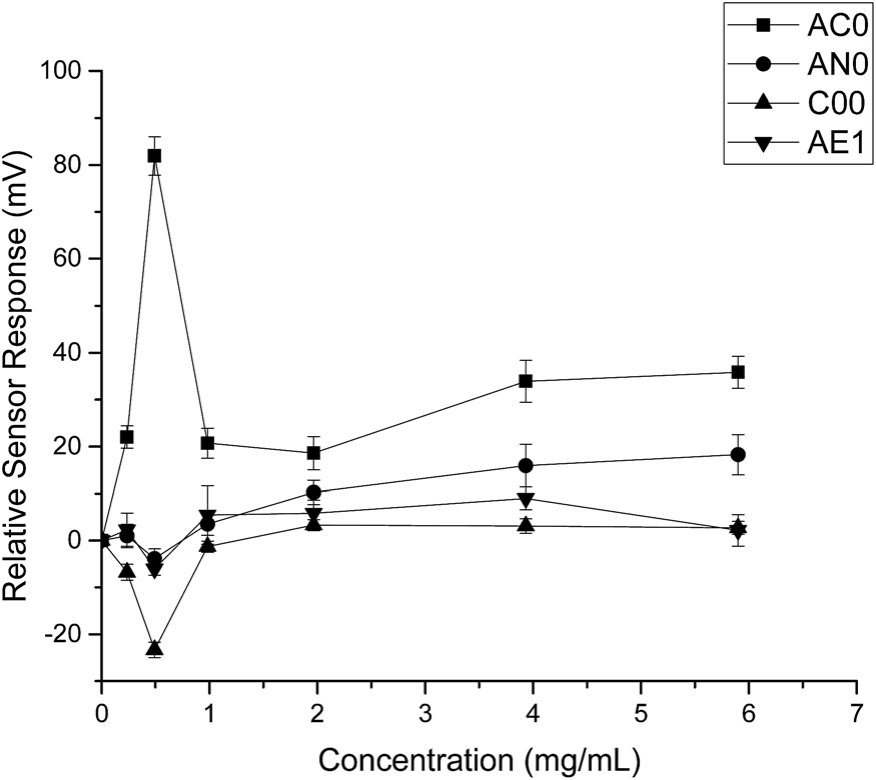
Sensor response curve for 2-(hydroxypropyl)-β-cyclodextrin showing normalised sensor response as a function of concentration (*n* = 3, mean ± S.D.).

When HP-β-CyD was assessed, both the absolute sensor responses and the concentration dependencies were weaker as compared to those of ranitidine hydrochloride (Fig. 3). Anomalies in sensor outputs were observed at 0.492 mg ml^−1^ (0.356 mM); SB2AC0 showed a sharp increase at these data points whereas the other three sensors displayed a fall in sensor responses. These responses also failed to depict clear dependency with concentrations of the complexing agent. For example, the sensor outputs from 0.983 mg ml^−1^ (0.713 mM) to 5.90 mg ml^−1^ (4.275 mM) of HP-β-CyD remained relatively constant without showing significant increase or decrease. A plausible explanation could be that the sweet tasting HP-β-CyD was difficult to characterize in a consistent manner by the bitterness sensors.^25^ It may also be an indication that the TS-5000Z taste sensing system is less effective in detecting non-ionic substances such as HP-β-CyD which has a neutral charge.^26^ As the measurement principal is potentiometric, the detection of ranitidine hydrochloride is comparatively enhanced due to increased conductivity associated with the cationic molecule.^27^

### Assessment of quinine hydrochloride dihydrate alone

Quinine hydrochloride dihydrate was chosen as the reference bitter model compound^28^ so that the taste of ranitidine hydrochloride could be expressed in relation to it on a PCA score plot. According to the European Pharmacopoeia, quinine hydrochloride has a bitterness value of 200 000 while ranitidine hydrochloride has a value of 100 000.^29^ A value of 200 000 means that 1 g of the substance diluted in 2001 parts of water still has a bitter taste. This implies that quinine hydrochloride dihydrate has a stronger bitter taste than ranitidine hydrochloride.

**Fig. 4:**
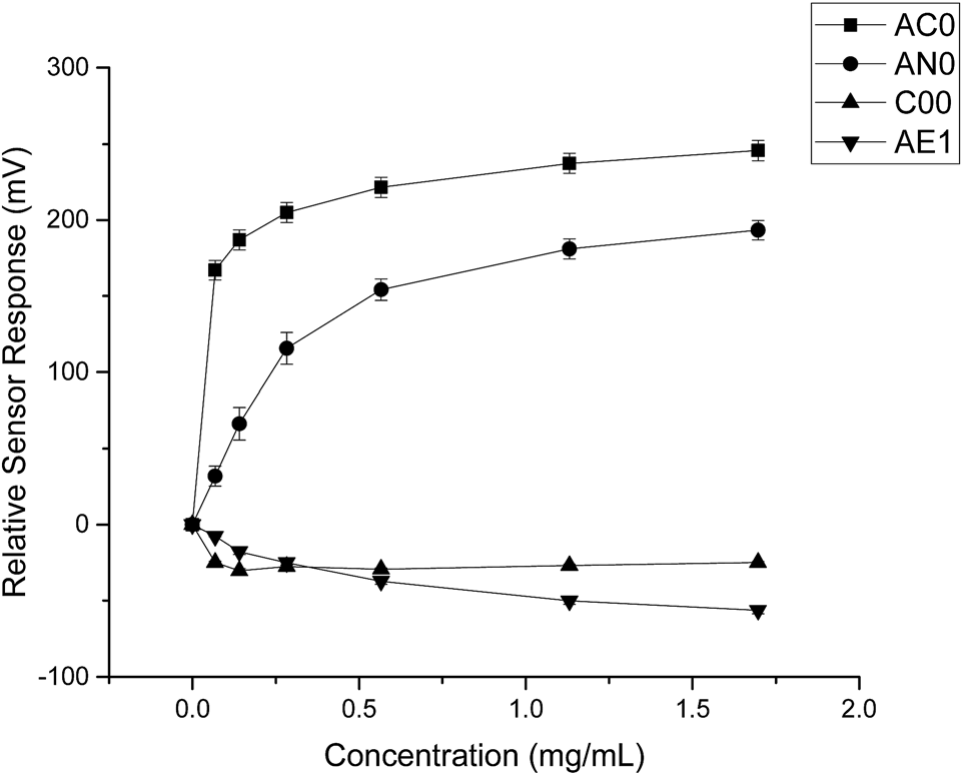
Sensor response curve for quinine hydrochloride dihydrate showing normalised sensor response as a function of concentration (*n* = 3, mean ± S.D.).

By comparing the data obtained from both substances, it was evident that quinine hydrochloride dihydrate elicited greater sensor responses compared to ranitidine hydrochloride (Fig. 4). For example, based on sensor SB2AC0, the sensor responses achieved at 1.50 mg ml^−1^ (4.275 mM) of ranitidine hydrochloride was 67 mV while the sensor response was 246 mV at 1.697 mg ml^−1^ (4.275 mM) of quinine hydrochloride dihydrate. The same was shown in sensor SB2AN0 where the sensor responses at 4.275 mM of ranitidine hydrochloride and quinine hydrochloride dihydrate were 56 mV and 193 mV correspondingly. The other dissimilarity between these two substances in terms of sensor response was observed in sensor SB2C00. Previously for ranitidine hydrochloride, sensor SB2C00 displayed a positive correlation between concentration and sensor response (Fig. 2); however, a negative correlation was presented with quinine hydrochloride dihydrate. Astringency sensor SB2AE1, on the other hand, showed similar responses for both drug substances. This comparison therefore allows us to both ascertain that, while nevertheless bitter tasting, ranitidine HCl is less so than quinine hydrochloride and also shows agreement with the literature assessment of their relative bitterness values.

### Principal component analysis of pure materials

**Fig. 5:**
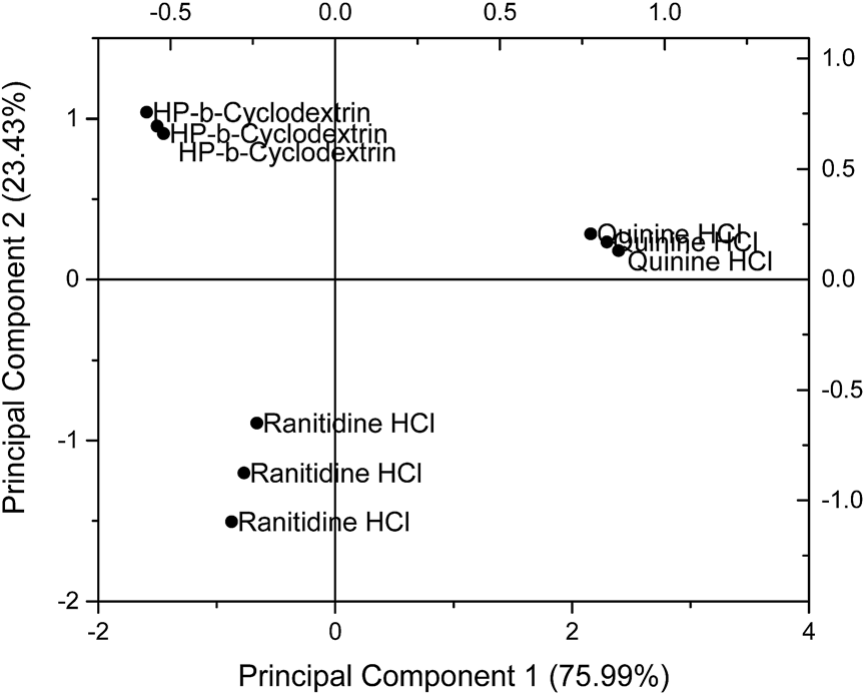
Principal component analysis (PCA) comparing sensor outputs of ranitidine hydrochloride with those of quinine hydrochloride dihydrate and HP-β-CyD using the following sensors: SB2AC0, SB2AN0, SB2C00 and SB2AE1.

Principal component analysis (PCA) was used to reduce the multidimensional space (*i.e.* responses from four independent sensors) without losing information. The drug sensor responses, together with that of HP-β-CyD, are presented on a PCA score plot (Fig. 5). The concentrations of ranitidine hydrochloride, quinine hydrochloride dihydrate and HP-β-CyD represented in this plot are of equal molarities (4.275 mM), corresponding to 1.50 mg ml^−1^, 1.697 mg ml^−1^ and 5.90 mg ml^−1^ respectively. In PCA, the dataset is projected onto the space spanned by the vectors (principal components) that correspond to the maximum variance of the dataset. Using PCA the most important information contained in the raw data could be transformed into the first principal component (PC-1) and the second most important is transformed into the second principal component (PC-2). Plotting of PC-1 *versus* PC-2 gives a map which allows the assessment of similarities and differences between different samples. Differences between samples were assessed by determining the Euclidean distance between them after multivariate data analysis. The greater the Euclidean difference between samples, the greater the difference in taste response.^21^

On the PCA map, bitter tasting quinine hydrochloride dihydrate and ranitidine hydrochloride were positioned on the right hand side and bottom left hand side respectively. HP-β-CyD, representing a benign tasting formulation, was located at the top left corner of the map. The Euclidean distance (*i.e.* the distance between two points on the map) between ranitidine hydrochloride and HP-β-CyD was calculated to be 2.29 while the distance between quinine hydrochloride dihydrate and HP-β-CyD was 3.87, indicating a stronger bitter taste for the latter drug which is consistent with the literature. The plot also demonstrates the ability of the taste sensing system to differentiate the two different bitter tasting drug substances as distinguished by their respective positions on the score plot. The experiments and associated plots were repeated in their entirety and both the plots and Euclidean distances were found to be similar.

### Taste-masked formulations

PCA was employed to examine the taste masking capabilities of HP-β-CyD on ranitidine hydrochloride by examining the responses for mixes of the two substances. The Euclidean distances between these formulations and ranitidine hydrochloride at each of the six concentration points were calculated to better assess taste masking efficiencies. As presented in Table 1, the distance from ranitidine hydrochloride generally increased from formulation with 1 : 1 molar ratio to formulation with 1 : 5 molar ratio, indicating that as the molar ratio of HP-β-CyD increased, the taste masking efficiency similarly increased; this is of interest in that from this data the optimal molar ratio is not simply 1 : 1.

**Table 1:** Euclidean distances of the taste-masked formulations from ranitidine hydrochloride at each individual concentration point. Molar ratios refer to drug: HP-β-CyD

Concentration of ranitidine hydrochloride (mg ml^−1^)	Euclidean distance from formulation to pure ranitidine hydrochloride				
	1 : 1 molar ratio	1 : 2 molar ratio	1 : 3 molar ratio	1 : 4 molar ratio	1 : 5 molar ratio
0.06	3.23	2.91	3.21	3.85	4.44
0.125	1.09	3.36	3.71	3.88	4.24
0.25	1.20	2.25	3.32	3.68	3.69
0.50	1.05	2.20	3.19	3.89	3.63
1.00	0.99	2.34	2.90	3.61	3.81
1.50	1.29	2.39	2.54	3.85	3.94

**Fig. 6:**
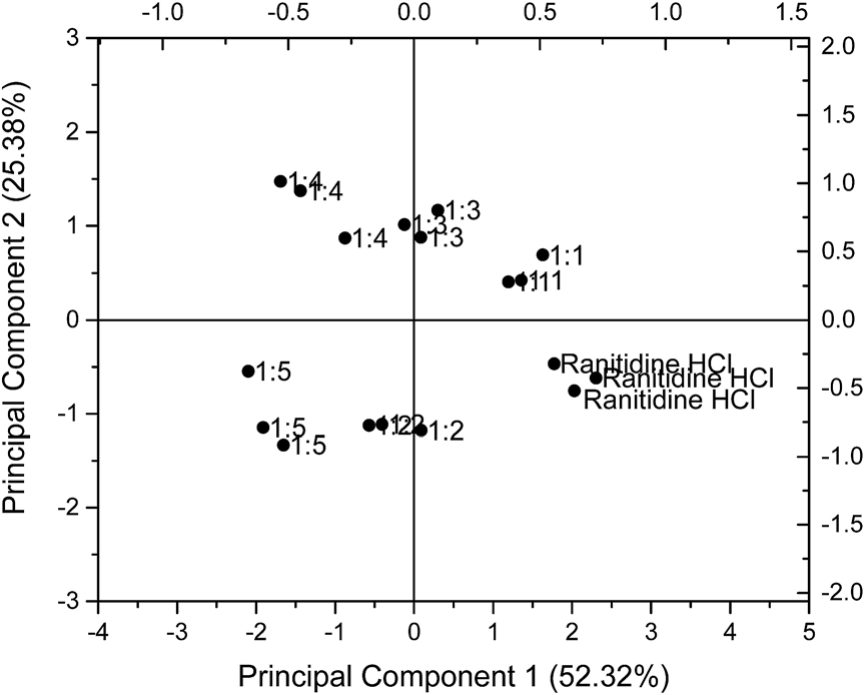
Principal component analysis (PCA) representing the influence of taste-masked formulations with different molar ratios (1 : 1, 1 : 2, 1 : 3, 1 : 4 and 1 : 5) of drug : HP-β-CyD on taste properties of 1.50 mg ml^−1^ (4.275 mM) ranitidine hydrochloride generated using the sensors SB2AC0, SB2ANO, SB2C00 and SB2AE1.

The results for 1.50 mg ml^−1^ (4.275 mM) ranitidine hydrochloride were chosen for illustration and presented on a PCA map in Fig. 6. On the PCA map, data points for ranitidine hydrochloride are located near the bottom right hand corner. It is observed that with increasing molar ratio of HP-β-CyD to ranitidine hydrochloride, the formulations were situated further away from the pure drug substance. The 1 : 1 molar ratio formulation has a Euclidean distance of 1.29, 1 : 2 molar ratio formulation has a value of 2.39, 1 : 3 molar ratio formulation has a value of 2.54 while 1 : 4 and 1 : 5 molar ratio formulations have values of 3.85 and 3.94 respectively. However, it is notable (Table 1) that the Euclidean distance of taste-masked formulations from 0.06 mg ml^−1^ (0.171 mM) of ranitidine hydrochloride did not follow this trend, with inconsistent results with molar ratio noted. These inconsistencies were anticipated as sensor response anomalies were previously detected at 0.06 mg ml^−1^ ranitidine hydrochloride and might have affected the analysis of sensor outputs.

In addition, it is observed that the Euclidean distance did not show significant increase in value at the formulations with 1 : 4 and 1 : 5 molar ratios; this may imply a maximum ratio for taste masking effectiveness, beyond which there is no advantage in raising the HP-β-CyD concentration. This is potentially highly useful in that the biosensor approach may give an indication to the formulator of maximum taste masking efficiency when using a relatively expensive masking excipient.

### NMR studies

**Fig. 7:**
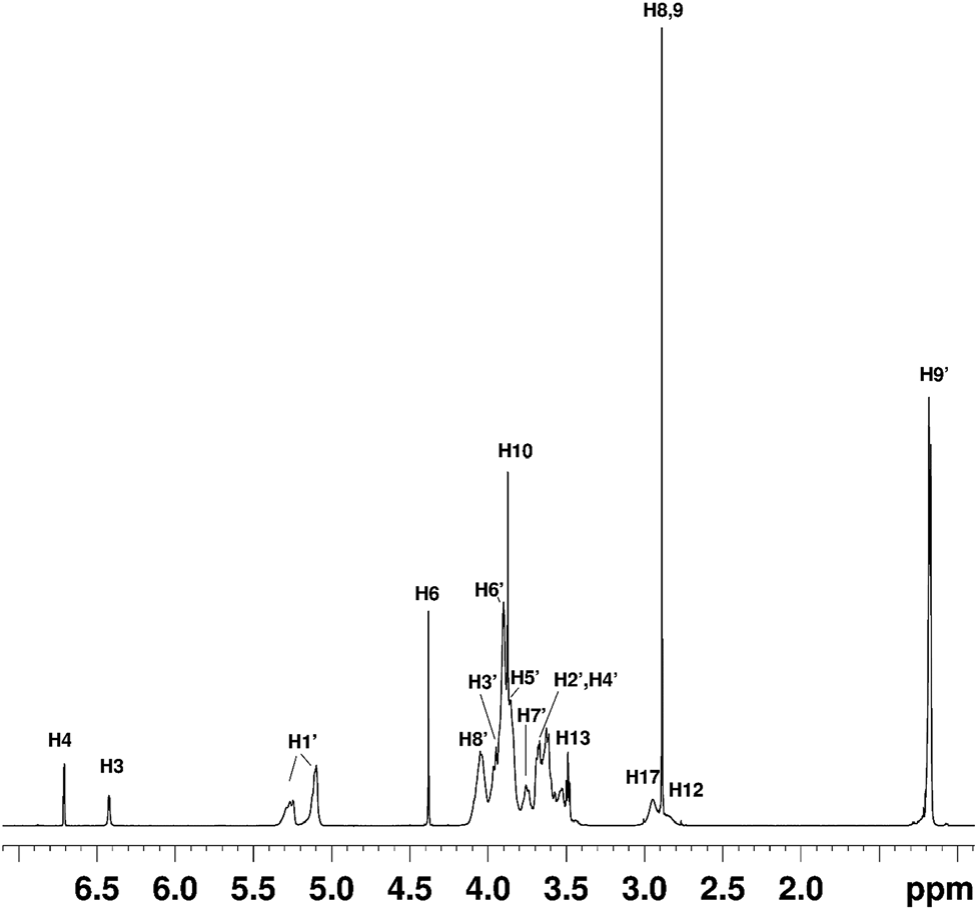
The ^1^H NMR spectrum of the HP-β-CyD : ranitidine hydrochloride mixture at *r* = 0.5. The chemical shifts of ranitidine hydrochloride are given in Table S5.†

NMR studies produced more supporting evidence for the inclusion of ranitidine hydrochloride into the central cavity of HP-β-CyD. It should be noted that (2-hydroxypropyl)-β-cyclodextrin used in this work is not structurally homogeneous, with the average degree of substitution reported as 0.5–1.3 unit of 2-hydroxypropyl (C_3_H_7_O) per glucose unit by the manufacturer. From the integral intensities of the signals between 4.94–5.50 ppm (H1′) and 0.88–1.50 (H9′) in the ^1^H NMR spectrum, the degree of substitution is estimated as 0.71 ± 0.02 for the sample of (2-hydroxypropyl)-β-cyclodextrin used in this work. Due to random substitution at different glucose ring positions, the ^1^H spectrum of HP-β-CyD is considerably more complex than that of β-CyD. As can be seen from Fig. 7, in the high-frequency region where proton H1′ resonates at 5.09 ppm in β-CyD,^30^ we observe signals between 5.09–5.31 ppm appearing as two sets of overlapping multiplets with centres of mass at 5.11 and 5.27 ppm due to protons H1′ with (or without) 2-hydroxypropyl substituents in different positions. The signal at 5.11 ppm can be attributed to glucose rings with no HP substituents or with HP substituents remote from the H1′ proton, thus causing only very small changes of the chemical shift (0–0.03 ppm) compared to that in β-CyD.^30^ Glucose rings with HP substituents in the vicinity of proton H1′ are likely to cause larger shift of H1′, and therefore the signal at 5.27 ppm is attributed to H1′ protons of glucose rings with HP substituents in the vicinity of proton H1′. For example, the HP substitution at C2 of the glucose ring is estimated to lead to the increase of the chemical shift of proton H1′ by 0.23 ppm using ACD/I-Lab database.

**Fig. 8:**
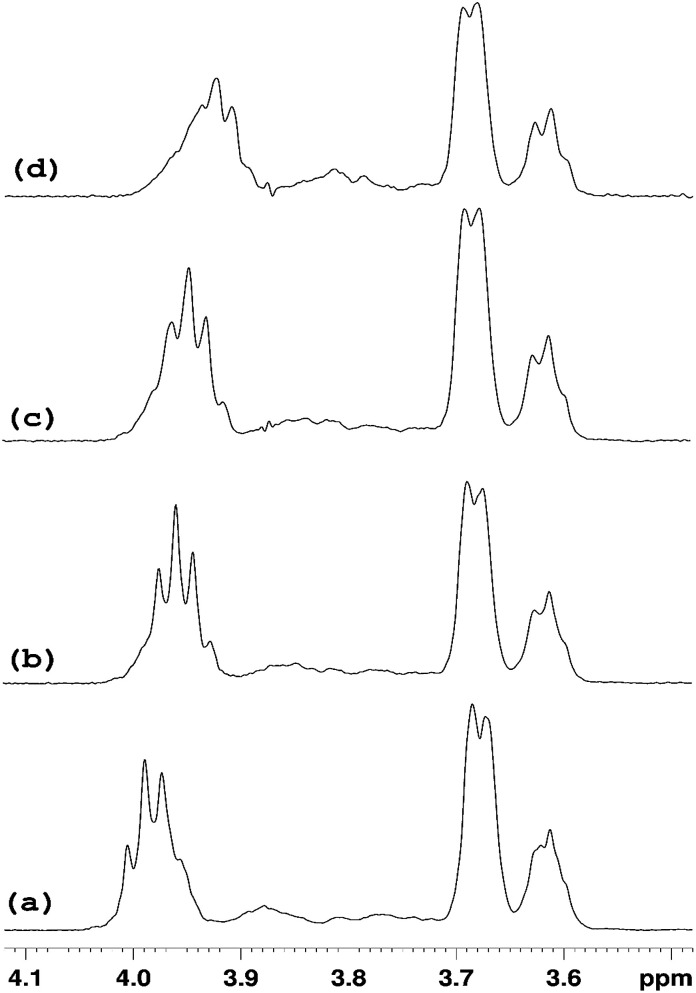
Selective TOCSY ^1^H NMR spectra of pure HP-β-CyD and HP-β-CyD with increasing proportions of ranitidine hydrochloride, with the selective pulse applied at the position of H1′ at 5.11 ppm. A gradual change in the position of the triplet H3′ signal at 3.99 ppm in (a) is observed on increasing proportions of ranitidine hydrochloride. The spectrum of pure HP-β-CyD is shown in (a). Spectra of (b), (c) and (d) are those of hydrochloride mixtures at 0.7, 0.5 and 0.3 respectively.

In order to simplify the spectrum selective TOCSY experiments were employed. The selective pulse of the TOCSY pulse sequence was applied at 5.11 ppm, which is narrower than that at 5.37 ppm, hence providing better selectivity for the identification of other glucose protons from the rings in which the H1′ proton resonates at 5.09–5.12 ppm. From the spectra shown in Fig. 8, chemical shifts of glucose ring protons were determined.

The chemical shifts of HP-β-CyD protons in the presence of increasing amounts of ranitidine hydrochloride is shown in Table S4.† In order to assess the effect of the inclusion of ranitidine hydrochloride in HP-β-CyD, the change in chemical shifts (Δ*δ*) in the complex (*δ*_complex_) relative to the chemical shifts of the same protons in the free components (*δ*_free_) was followed, where Δ*δ* is defined as (*δ*_complex_ − *δ*_free_). The complexation-induced chemical shifts (Δ*δ*) were the most significant at H3′ proton of HP-β-CyD (see numbering of HP-β-CyD protons in Fig. 1). At *r* = 0.7 (70% molar ratio of HP-β-CyD; see Table S3†), the shift change was −30 ppb (Table S4†). At *r* = 0.5 (50% HP-β-CyD), the chemical shift change was −43.3 ppb, while the value was −66.8 ppb at *r* = 0.3 (30% HP-β-CyD). A greater magnitude of change in the chemical shift for H3′ protons of HP-β-CyD compared to other protons of HP-β-CyD could be due to proximity of protons H3′ to the aromatic furan ring of ranitidine, which is likely to cause larger chemical shift changes than other functionalities of ranitidine due to the aromatic ring current effects (*i.e.* shielding with the decrease of ^1^H chemical shifts, if proton H3′ is placed above the furan ring, as observed experimentally). It is also interesting to note (see Fig. 8 and Table S4†) that the level of H3′ shift change was greatest at the lowest ratio (*r*) of polymer to drug; this is consistent with expectations that the higher drug loading would result in a greater occupancy of the binding site on the HP-β-CyD. In principle, an exchange model is also consistent with this explanation: there are H3′ sites with and without ranitidine molecules nearby. The chemical shift we see for H3′ of the host is averaged between that of free H3′ and bound H3′ as a result of fast diffusion of the guest molecules inside (or in and out of) the host cavities. So, at higher loadings we will see larger chemical shift change for H3′ of the host molecules, simply because at any instant we have more H3′⋯guest pairs than H3′⋯no guest.

^1^H and ^13^C chemical shifts of pure ranitidine hydrochloride and in HP-β-CyD : ranitidine hydrochloride mixtures are included in Table S5.† The largest chemical shift changes were observed for protons H4 and H6, as well as for carbons C18, C12, C2 and C10. Such changes could be attributed to proximity of corresponding atoms to HP-β-CyD molecules or the changes of the conformation of the ranitidine hydrochloride inside the HP-β-CyD cavities compared to its preferred conformation in D_2_O solution.

**Fig. 9:**
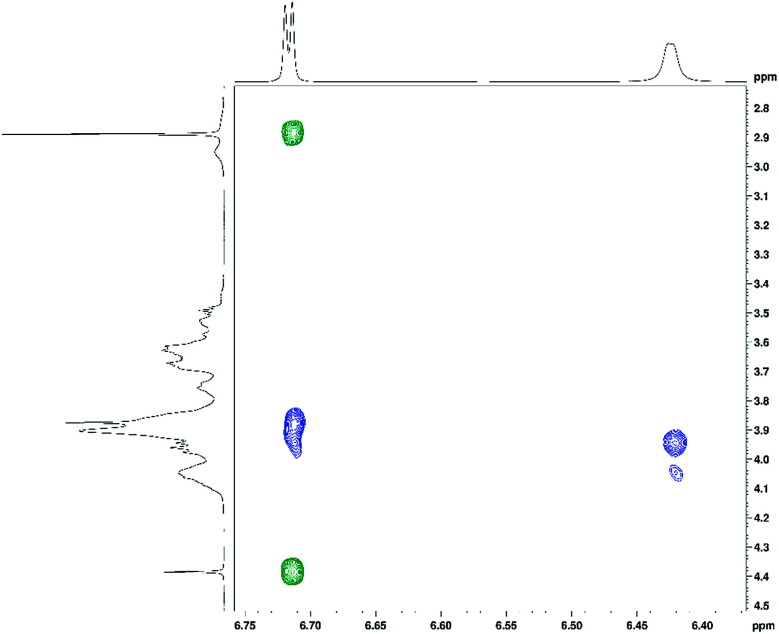
The 2D NOESY spectrum of the HP-β-CyD : ranitidine hydrochloride mixture with *r* = 0.7, showing positive (in green) intramolecular and negative (in blue) intermolecular NOEs for ranitidine furan protons.

In addition to ^1^H and ^13^C chemical shift changes of host and guest species, we have also sought direct evidence from NMR experiments in order to confirm complexation of ranitidine and HP-β-CyD molecules. Fig. 9 shows nuclear Overhauser effects (NOEs) of furan protons of ranitidine in the two-dimensional (2D) NOESY spectrum of HP-β-CyD : ranitidine hydrochloride mixture with *r* = 0.7. Positive (shown in green) and negative (shown in blue) NOEs are observed, which are usually characteristic for small and high molecular weight species, respectively. The observation of intermolecular negative NOEs between ranitidine furan protons (at 6.41 and 6.68 ppm) and HP-β-CyD cavity protons H3′ and H5′ (resonating at 3.96 and 3.88 ppm, respectively) confirms the inclusion of ranitidine molecules inside the HP-β-CyD cavity.

A weaker intermolecular negative NOE is also observed between the ranitidine furan proton H3 (at 6.42 ppm) and the HP-β-CyD proton H8′ (at 4.05 ppm). Similar intermolecular NOEs were also observed for other complexes of HP-β-CyD : ranitidine hydrochloride. Comparison of the volume integral of the intermolecular NOE at ∼3.9–6.7 ppm relative to that of the intramolecular NOE at ∼2.9–6.7 ppm, showed the decrease of the intensity of the intermolecular NOE on decreasing the ratio (*r*) of polymer to drug: 1.5 in *r* = 0.7, 1.1 in *r* = 0.5 and 0.5 in *r* = 0.3. This, in principle, may suggest stronger binding of the guest at the highest ratio of polymer to drug.

### Molecular modelling

**Fig. 10:**
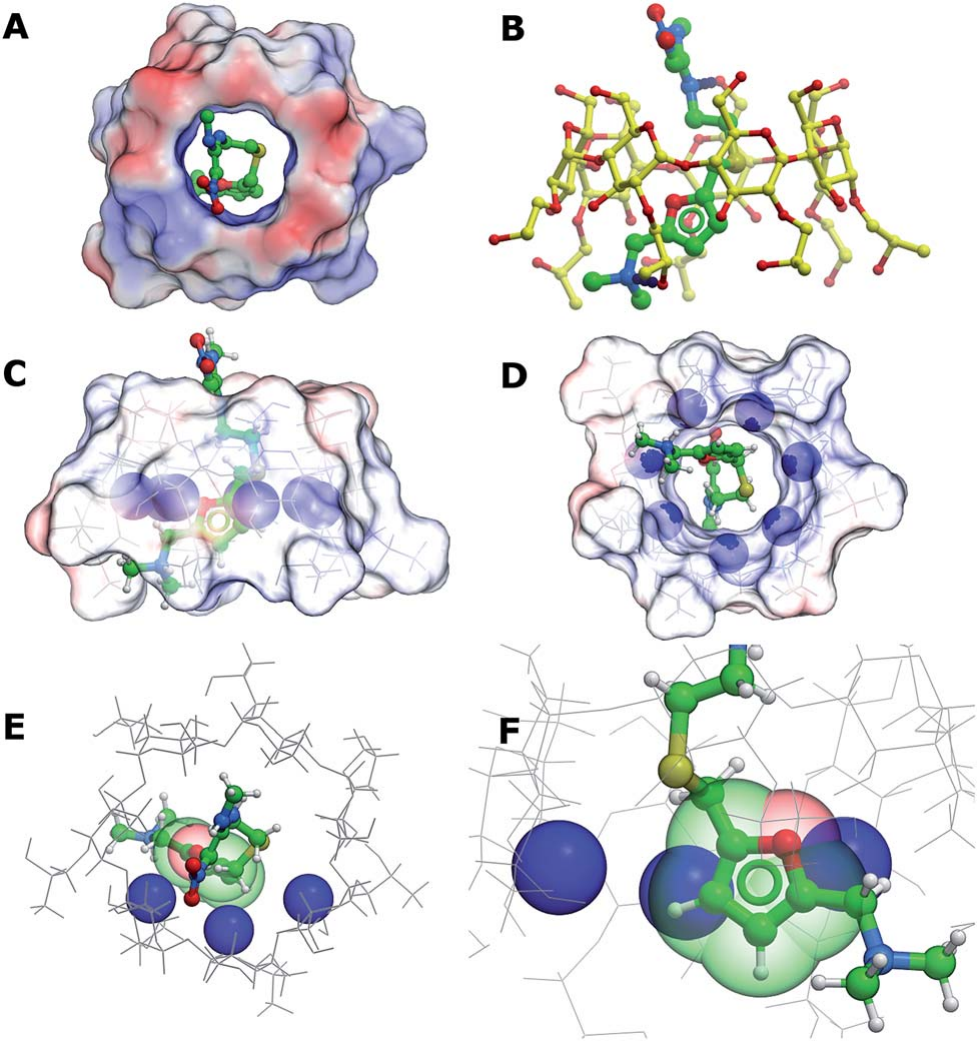
(A) Top view of the electrostatic surface of the donut shaped HP-β-CyD. (B) Ranitidine (green) binds in the central cavity, making hydrogen bonds with 6′-OH and 8′-OH groups of HP-β-CyD (yellow). (C) Side and (D) end view of the positions of H3′ protons (blue sphere) in HP-β-CyD (E and F) top and side views of the spatial proximity between H3′ protons and the furan ring (green spheres) in ranitidine.

In order to better understand the interactions between (2-hydroxypropyl)-β-cyclodextrin and ranitidine, molecular docking studies were performed. The central cavity of the HP-β-CyD has a diameter of 13.2 Å, and is hence large enough to allow ranitidine to penetrate into the interior (Fig. 10A). The dimethylamino group (N7) at one end of the drug molecule and the nitrogen atom in the diamine group (N14) at the other form direct hydrogen bonds with oxygen atoms at 6′-OH and 8′-0H of the HP-β-CyD (Fig. 10B). The binding energy was calculated to be −19.95 kcal mol^−1^. Ranitidine docks with its furan ring in close proximity to H3′ (Fig. 10C–F). The molecular complex between HP-β-CyD and ranitidine is therefore consistent with results from NMR experiments, which indicated that the largest chemical shifts are for H3′ proton and suggested that these are associated with a close proximity of the furan ring of the drug to this proton. Our modelling studies fully support this, suggesting ring current effects arising from the close proximity of the furan ring of the ranitidine molecule.

## Conclusions

The present study demonstrates that taste masking efficiency can be examined by sensor response measurements. Using the TS-5000Z taste sensing system, sensor outputs were obtained from various taste-masked formulations and further analysed using PCA. The results indicated that the Euclidean distance from the pure drug substance (ranitidine hydrochloride) increased from the formulation with 1 : 1 molar ratio to formulation with 1 : 5 molar ratio. This provided strong evidence that with increasing molarity of HP-β-CyD in a formulation, the bitter taste of the formulation decreased. These results were further supported by the NMR studies. The ^1^H-NMR spectra showed significant complexation induced shifts when increasing proportions of ranitidine hydrochloride were added to HP-β-CyD. The H3′ protons of HP-β-CyD displayed the greatest magnitude of change in terms of chemical shifts as compared to other protons of the glucose ring; this proton, as well proton H5′ are oriented inwards inside the β-CyD cavity (as confirmed here using modelling studies). Further NMR measurements revealed intermolecular NOEs between internal protons H3′ and H5′ of the HP-β-CyD cavity and furan protons of ranitidine hydrochloride, thus confirming their spatial proximity. A plausible mode of structural interaction that is consistent with the experimental data is also suggested using molecular docking studies, predicting hydrogen bond interactions between two nitrogen groups at either end of the molecule with hydroxyl oxygen groups of HP-β-CyD, plus the docking studies also indicated an interaction between the furan group of the drug with the internal H3′ proton of the HP-β-CyD, in agreement with the NMR studies. The biosensor system used to assess taste is primarily a potentiometric approach, hence it can be seen that molecular interaction between the drug and HP-β-CyD can be reasonably expected to reduce the electrode potential of the drug leading to a lower measurement for bitterness.

The study therefore indicates that the interaction between the drug and complexing agent (confirmed using NMR and docking studies) is reflected by a change in taste, with an associated decrease in bitterness predicted. This is significant in that it provides the first stage for the development of a screening approach for complexation that could allow identification of the most promising formulations at an early stage, thus saving considerable time and expense. The fact that the observed biosensor responses may be supported by associated structural studies provides a means of mechanistically supporting, and potentially predicting, the taste assessment measurements.

The obvious question remains of how specifically the biosensor information translates to the biological environment, but given the well-known physiological taste masking capability of cyclodextrins it is entirely reasonable to suggest firstly a clear causality between the complexation demonstrated here and the reduction in taste suggested by the biosensor system and secondly a putative causality between the complexation configuration and the reduction in taste *in vivo*. This is significant in that it develops the discussion on how molecular configuration (and shielding thereof) relates to human taste, a much larger issue about which as yet little is known. Overall therefore the study has provided a mechanistic link between complexation of an API (ranitidine hydrochloride) with HP-β-CyD and the associated taste as measured using a biosensor system.

## Conflicts of interest

There are no conflicts to declare.

## Supplementary Material

RA-008-C7RA11015D-s001
