# Supplementary material for: Evaluation of the taste-masking effects of (2-hydroxypropyl)-β-cyclodextrin on ranitidine hydrochloride; a combined biosensor, spectroscopic and molecular modelling assessment
Source: RSC Adv. 2018 Jan 17;8(7):3564–73. doi: 10.1039/c7ra11015d (PMC9077696; doi:10.1039/c7ra11015d)
Supplement: RA-008-C7RA11015D-s001 [file RA-008-C7RA11015D-s001.pdf]

Electronic Supplementary Material (ESI) for RSC Advances.
This journal is © The Royal Society of Chemistry 2018

Supplementary Information

Table S1 Concentration series of ranitidine hydrochloride, quinine hydrochloride dihydrate and HP-β-CyD in equal molarity.

**Drug substance**	**Concentration (mg/ml)**					
Ranitidine HCl	0.06	0.125	0.25	0.50	1.00	1.50
Quinine HCl dihydrate	0.068	0.141	0.283	0.566	1.131	1.697
HP-β-CyD	0.236	0.492	0.983	1.967	3.933	5.90
Molarity (mM)	0.171	0.356	0.713	1.425	2.850	4.275

Table S2 Concentration of HP-β-CyD to be mixed with ranitidine hydrochloride to prepare taste-masked formulations of five different molar ratios (1:1, 1;2, 1:3 1:4 and 1:5).

**Concentration of ranitidine hydrochloride (mg/ml)**	**Concentration of HP-β-CyD (mg/ ml) in taste-masked formulation**				
	**1:1 molar ratio**	**1:2 molar ratio**	**1:3 molar ratio**	**1:4 molar ratio**	**1:5 molar ratio**
0.06	0.236	0.472	0.708	0.944	1.180
0.125	0.492	0.983	1.475	1.967	2.458
0.25	0.983	1.967	2.950	3.933	4.916
0.50	1.967	3.933	5.90	7.866	9.833
1.00	3.933	7.866	11.80	15.73	19.67
1.50	5.90	11.80	17.70	23.60	29.50

Table S3 Proportions of HP-β-CyD and ranitidine hydrochloride used in sample preparation for the NMR studies.

**HP-β-CyD (mM)**	**Ranitidine hydrochloride (mM)**	**Sum (mM)**	**Ratio (r)**
12	28	40	0.3
20	20	40	0.5
28	12	40	0.7

Table S4 ^{1}H NMR chemical shifts (δ, ppm) of pure HP-β-CyD and HP-β-CyD:Ranitidine hydrochloride mixtures extracted from selective 1D TOCSY spectra. r = 0.3, 0.5 and 0.7 signify 30%, 50% and 70% of HP-β-CyD in HP-β-CyD:Ranitidine hydrochloride mixtures, respectively.[a]

Proton	Chemical shifts, δ (ppm)				Chemical shift differences, δ (ppb)		
	HP-β-CyD	r = 0.3	r = 0.5	r = 0.7	r = 0.3	r = 0.5	r = 0.7
H1'	5.1071	5.1016	5.1036	5.1045	-5.5	-3.5	-2.6
H2'	3.6782	3.6876	3.684	3.6823	9.4	5.8	4.1
H3'	3.9895	3.9227	3.9462	3.9595	-66.8	-43.3	-30
H4'	3.6128	3.6118	3.6119	3.6124	-1	-0.9	-0.4

[a]*Protons H5' and H6' of the glucose ring show overlapping multiplets at 3.81 and 3.88 ppm, respectively. Due to their overlap and small intensities in 1D TOCSY spectra, no attempt was made to follow the concentration dependence for these protons.*

Table S5 ^{1}H and ^{13}C **c**hemical shifts of pure ranitidine hydrochloride and in HP-β-CyD:Ranitidine hydrochloride mixtures. Ratios r = 0.3, 0.5 and 0.7 signify 30%, 50% and 70% of HP-β-CyD in HP-β-CyD:Ranitidine hydrochloride mixtures, respectively.

Atom	Chemical shifts, δ (ppm)				Chemical shift differences, Δδ (ppb)		
	Ranitidine	r = 0.3	r = 0.5	r = 0.7	r = 0.3	r = 0.5	r = 0.7
H4	6.6843	6.7009	6.7103	6.7167	16.6	26	32.4
H3	6.4142	6.4202	6.4227	6.4244	6	8.5	10.2
H6	4.3648	4.3756	4.3813	4.3849	10.8	16.5	20.1
H10	3.8704	3.8739	3.8746	3.8751	3.5	4.2	4.7
H13	3.481	3.4871	3.4897	3.4915	6.1	8.7	10.5
H17[a]	2.94						
H12[a]	2.92						
	2.82						
H8,9	2.8859	2.8887	2.8891	2.8895	2.8	3.2	3.6
C15	156.66	156.65	156.65	156.66	-10	-10	0
C5	154.81	154.77	154.75	154.73	-40	-60	-80
C2	143.83	143.91	143.97	144.01	80	140	180
C4	116.2	116.22	116.23	116.24	20	30	40
C3	110.07	110.06	110.06	110.06	-10	-10	-10
C18[a]	101.37	101.24	101.17	101.12	-130	-200	-250
C6	53.44	53.42	53.41	53.4	-20	-30	-40
C8,9	42.53	42.55	42.56	42.56	20	30	30
C13[a]	41.99	41.96	41.94	42	-30	-50	10
	40.28	40.34	40.36	40.39	60	80	110
C12[a]	31.45	31.48	31.46	31.53	30	10	80
	30.27	30.35	30.45	30.52	80	180	250
C17	29.09	29.16	29.16	29.16	70	70	70
C10	28.15	28.21	28.25	28.29	60	100	140

[a]*The observation of two relatively broad peaks with a 1:1 ratio for carbons C12 and C13 is attributed to the fact that ranitidine exists as a 1:1 mixture of E or Z isomers, depending on the orientation of the nitroethylenediamine group.*[31] *The exchange between these two isomers is in the slow regime in the ^{13}C NMR timescale, while it is in the intermediate regime in the ^{1}H NMR timescale leading to very broad signals for H12 and H17 in the ^{1}H NMR spectrum. No signal for H18 is observed and C18 is observed as a 1:1:1 triplet with a 28.5 Hz separation, which suggests the substitution of H18 by a deuterium atom from D_2O, used as solvent in NMR measurement*
